# Genetic variability in the glucocorticoid pathway and treatment outcomes in hospitalized patients with COVID-19: a pilot study

**DOI:** 10.3389/fphar.2024.1418567

**Published:** 2024-07-29

**Authors:** Patricija Štampar, Tanja Blagus, Katja Goričar, Petra Bogovič, Gabriele Turel, Franc Strle, Vita Dolžan

**Affiliations:** ^1^ Pharmacogenetics Laboratory, Institute of Biochemistry and Molecular Genetics, Faculty of Medicine, University of Ljubljana, Ljubljana, Slovenia; ^2^ Department of Infectious Diseases, University Medical Centre Ljubljana, Ljubljana, Slovenia

**Keywords:** dexamethasone, methylprednisolone, glucocorticoid pathway, polymorphism, treatment outcome, COVID-19

## Abstract

**Introduction:** Corticosteroids are widely used for the treatment of coronavirus disease (COVID)-19. Genetic polymorphisms of the glucocorticoid receptor, metabolizing enzymes, or transporters may affect treatment response to dexamethasone. This study aimed to evaluate the association of the glucocorticoid pathway polymorphisms with the treatment response and short-term outcomes in patients with severe COVID-19.

**Methods:** Our pilot study included 107 hospitalized patients with COVID-19 treated with dexamethasone and/or methylprednisolone, genotyped for 14 polymorphisms in the glucocorticoid pathway.

**Results:** In total, 83% of patients had severe disease, 15.1% had critical disease and only 1.9% had moderate disease. *CYP3A4* rs35599367 was the major genetic determinant of COVID-19 severity as carriers of this polymorphism had higher risk of critical disease (OR = 6.538; 95% confidence interval = 1.19–35.914: *p* = 0.031) and needed intensive care unit treatment more frequently (OR = 10; 95% CI = 1.754–57.021: *p* = 0.01). This polymorphism was also associated with worse disease outcomes, as those patients had to switch from dexamethasone to methylprednisolone more often (OR = 6.609; 95% CI = 1.137–38.424: *p* = 0.036), had longer hospitalization (*p* = 0.022) and needed longer oxygen supplementation (*p* = 0.040). Carriers of *NR3C1* rs6198 polymorphic allele required shorter dexamethasone treatment (*p* = 0.043), but had higher odds for switching therapy with methylprednisolone (OR = 2.711; 95% CI = 1.018–7.22: *p* = 0.046). Furthermore, rs6198 was also associated with longer duration of hospitalization (*p* = 0.001) and longer oxygen supplementation (*p* = 0.001). *NR3C1* rs33388 polymorphic allele was associated with shorter hospitalization (*p* = 0.025) and lower odds for ICU treatment (OR = 0.144; 95% CI = 0.027–0.769: *p* = 0.023). *GSTP1* rs1695 was associated with duration of hospitalization (*p* = 0.015), oxygen supplementation and (*p* = 0.047) dexamethasone treatment (*p* = 0.022).

**Conclusion:** Our pathway-based approach enabled us to identify novel candidate polymorphisms that can be used as predictive biomarkers associated with response to glucocorticoid treatment in COVID-19. This could contribute to the patient’s stratification and personalized treatment approach.

## 1 Introduction

Severe Acute Respiratory Syndrome-Coronavirus 2 (SARS-CoV-2) first appeared in Wuhan, China, at the end of 2019 and rapidly spread worldwide, causing global pandemic of coronavirus disease 2019 (COVID-19) (Ferrara and Vitiello, 2021). While in the majority of individuals this viral infection occurs without or with mild symptoms, in a small proportion of infected individuals, the virus may cause more severe or critical form of COVID-19, ultimately leading to a fatal outcome (Samadizadeh et al., 2021). Upon SARS-CoV-2 infection, the body produces pro-inflammatory cytokines (IL-6, IL-12, IFN-γ) and chemokines (CXCL10, CCL2) in order to elicit immune response and protect the body from the infection (Solinas et al., 2020). In infected individuals who develop mild COVID-19, the level of these pro-inflammatory cytokines and chemokines is usually low. In contrast, in individuals who develop severe or critical COVID-19, their levels are often excessive. This strong and uncontrolled production of soluble inflammatory mediators is known as a “cytokine storm” and is responsible for the infiltration of immune cells in lungs, resulting in acute lung injury, hypoxemia and acute respiratory distress syndrome (ARDS) (Alexaki and Henneicke, 2021; Noreen et al., 2021). Besides the respiratory system, other organs may also be involved (Ferrara and Vitiello, 2021). Vaccination against SARS-CoV-2 reduces the risk of infection as well as the development of a more severe COVID-19, but the appropriate personalized treatment approaches for already infected individuals remain unclear (Kino et al., 2021). Managing “cytokine storm” seems to be a crucial step in COVID-19 treatment and thus suggests the usage of drugs, such as glucocorticoids (GCs) that attenuate the immune response with their anti-inflammatory properties (Solinas et al., 2020).

GCs had previously been used to manage ARDS caused by SARS-CoV in 2003 and Middle East Respiratory Syndrome-related coronavirus (MERS-CoV) in 2012 (Solinas et al., 2020; Kino et al., 2021). At the beginning of COVID-19 epidemic, the WHO did not recommend GSs as treatment option for patients with COVID-19, as GCs can prolong viral clearance (Solinas et al., 2020). Researchers in Oxford RECOVERY Trial investigated several different COVID-19 treatment options, including dexamethasone, lopinavir, ritonavir, azithromycin, and hydroxychloroquine. This trial showed that only dexamethasone in low doses (6 mg per day, up to 10 days) reduced COVID-19 associated deaths by one-fifth in patients with oxygen supplementation and by one-third in patients with mechanical ventilation therapy. However, no benefit of dexamethasone treatment was observed in patients with mild disease (Horby et al., 2021; Noreen et al., 2021).

Dexamethasone is a synthetic corticosteroid that has been used since 1960 for the therapy of various inflammatory conditions, such as systemic lupus, various types of arthritis, allergic conditions and skin conditions (Vohra et al., 2021). With its anti-inflammatory and immuno-suppressive properties, it helps attenuate the host’s excessive immune response to SARS-CoV-2 infections. Moreover, dexamethasone is highly potent and has a long plasma half-life (approximately 4–6 h), making it a favorable choice in the treatment of severe COVID-19 (Kino et al., 2021). It is primarily transported across the human body by serum albumin (Shabalin et al., 2020). Being a lipid-soluble molecule, dexamethasone can easily enter the target cell where it binds to the inactive glucocorticoid receptor (GR) (Gray et al., 2017). The receptor then undergoes conformational changes, dimerizes and translocates to the nucleus, where it acts as a transcriptional factor (Solinas et al., 2020; Alexaki and Henneicke, 2021), either inhibiting or inducing the expression of immune response genes (Gray et al., 2017; Kino et al., 2021). GRs are encoded by *NR3C1* gene and appear in two isoforms, GRα and GRβ, of which only GRα isoform can bind GCs (Gasic et al., 2018; Solinas et al., 2020). Dexamethasone is metabolized in the liver. In the phase 1 of biotransformation CYP3A4 and, to lesser extent CYP3A5, catalyze 6β-hydroxylation of dexamethasone (Bergmann et al., 2012; Vohra et al., 2021). In phase 2, glutathione S-transferases (GSTs) catalyze the conjugation of hydroxylated dexamethasone with glutathione (GSH). In phase 3, transporters such as ATP-binding cassette subfamily B member 1 (ABCB1) transport the resulting hydrophilic conjugates out of the cell (Esteves et al., 2021; Pahor et al., 2021).

Genetic polymorphisms of the GR, metabolizing enzymes, or transporters were reported to affect the treatment response to GCs (Bergmann et al., 2012; Association, 2013; Pahor et al., 2021). In particular, *NR3C1*, *CYP3A4*, and *CYP3A5* polymorphisms were shown to modulate the pharmacokinetics of dexamethasone (Vohra et al., 2021). Polymorphisms in *NR3C1*, *GSTs*, and *ABCB1* genes were also associated with response to GC treatment in different diseases (Pahor et al., 2021; Fishchuk et al., 2023). The aim of this study was to evaluate the associations of polymorphisms in the GC pathway with treatment outcomes in hospitalized Slovenian patients with COVID-19. The following treatment outcomes were investigated: disease severity, ICU treatment requirement, duration of hospitalization, duration of oxygen supplementation, duration of dexamethasone treatment and odds for switching from dexamethasone to methylprednisolone treatment.

## 2 Materials and methods

### 2.1 Patients and clinical data

This pilot study included patients with PCR confirmed COVID-19 that were at least 18 years old, and hospitalized at the Department of Infectious Diseases, University Medical Centre Ljubljana, between September 2020 and December 2021.

This study included only patients that were treated with GC. In the majority of patients included in the study, hypoxaemia was present on admission and was the reason (or one of the main reasons) for admission to hospital. In these patients dexamethasone in concentration of 6 mg per day was introduced at admission to hospital. However, in a part of patients hypoxemia occurred during hospitalisation—in these patients GC treatment was introduced after admission, i.e., at the time of detection of hypoxaemia. GC treatment lasted for 10 days, or for a shorter period if blood oxygen saturation improved earlier. Dexamethasone treatment was switched to methylprednisolone when a patient did not respond to treatment and required 100% oxygen, non-invasive ventilation or mechanical ventilation. In these patients 1 mg/kg methylprednisolone was introduced and then gradually decreased depending on the course of the disease (Salton et al., 2020; Yang et al., 2020).

The following clinical and demographic data were collected: (i) gender, (ii) age, (iii) BMI, (iv) presence of chronic diseases (diabetes, hyperlipidemia, arterial hypertension, heart failure and chronic lung disease), (iv) duration of dexamethasone treatment, (vi) duration of the hospitalization, (vii) duration of oxygen supplementation, (viii) switching dexamethasone for methylprednisolone treatment, (ix) treatment in intensive care unit (ICU), and (x) COVID-19 disease severity. Disease severity has been classified into four groups: a) mild disease was defined by condition without pneumonia, b) moderate disease was defined as condition with pneumonia but without requirement for oxygen supplementation and c) severe disease was defined by severe pneumonia with requirement for oxygen supplementation with up to 15 L per minute and d) critical disease was defined as condition with ARDS and required oxygen supplementation with more than 15 L per minute.

The primary outcomes investigated in patients treated for COVID-19 were duration of dexamethasone treatment and treatment switching from dexamethasone to methylprednisolone. The secondary outcomes were disease severity, duration of hospitalization, duration of oxygen supplementation and the need for ICU treatment.

This study was approved by the National Medical Ethics Committee of the Republic of Slovenia (No 0120-211/2020/7 and 0120-452/2021/3). The informed consent for the participation in the study was obtained from all patients.

### 2.2 Genetic polymorphisms selection

Based on previous studies of pharmacogenomics markers in glucocorticoid pathway (Bergmann et al., 2012; Sæves et al., 2012; Gasic et al., 2018; Pahor et al., 2021), we selected a total of 14 genetic variants that included single nucleotide polymorphisms (SNPs) and gene deletions: *NR3C1* rs6198, rs33388 and rs33389, *CYP3A4* rs35599367 and rs2740574, *CYP3A5* rs776746 and rs10264272, *GSTP1* rs1695 and rs1138272, *GSTM1* and/or *GSTT1* deletion and *ABCB1* rs1045642, rs1128503 and rs2032582. The characteristics of the investigated SNPs/gene deletions are presented in Supplementary Table S1.

### 2.3 DNA isolation and genotyping analysis

From all patients 6 mL of peripheral blood was collected in vials with ethylene diamine tetraacetic acid (EDTA) at admission to the hospital and stored at −20°C until DNA extraction. Genomic DNA was extracted using the commercial kit E. Z.N.A.^®^ SQ II Blood DNA Kit (Omega BioTek, Georgia, United States) according to the manufacturer’s instructions. Quality and quantity of isolated DNA has been verified spectrophotometrically and stored at 4°C until use. All samples were genotyped for the selected variants. All SNPs, except for *ABCB1* rs2032582, were genotyped using competitive allele specific PCR reaction (KASPar) (LGC Biosearch Technologies, Hoddesdon, United Kingdom) according to the manufacturer’s instructions. The triallelic *ABCB1* rs2032582 SNP was determined by multiplex PCR as previously described (Gasic et al., 2018). *GSTT1* and *GSTM1* gene deletions were determined by PCR as previously described (Krhin et al., 2016).

### 2.4 Statistical analysis

Central tendency and variability of continuous variables was described with median and 25th and 75th percentile range, which was reported in the results in the form: median, 25–75%. The distribution of categorical variables was described with frequencies. χ2-Test was used to examine the agreement of genotype frequencies with the Hardy-Weinberg equilibrium. Nonparametric Mann-Whitney *U* test or Kruskal–Wallis tests were used for analyses of effects of numerical data on selected outcomes and logistic regression was used to calculate odds ratio (OR) and 95% confidence interval (CI). ANCOVA test was used to adjust the genetic models for statistically significant clinical covariates such as the presence of chronic diseases, age, or gender when they were related to the studied outcomes. Haplotype analysis was conducted to evaluate the combined impact of several SNPs within the same gene. Haplotypes were reconstructed and analyzed together with studied outcomes with the Thesias program (Tregouet and Garelle, 2007). Only haplotypes with frequencies above 5% were considered in the analysis and the most common haplotype was used as a reference. All statistical tests were conducted using a two-sided approach. To address the issue of multiple comparisons and minimize the chances of false positive results, we applied the Bonferroni correction. For the analysis of genetic data, *p*-values equal to or below 0.0038 (0.05 divided by 13) were considered statistically significant, while *p*-values between 0.0038 and 0.0500 were regarded as nominally significant. These stringent thresholds were implemented to ensure robustness and minimize the likelihood of false positive results. For disease severity, this study had 80% power to detect ORs of 4.6 or more for a polymorphism with minor allele frequency of 0.30 and ORs of 5.9 or more for a polymorphism with minor allele frequency of 0.10 or 0.50. PS Power and sample size calculations, version 3.0. Was used for power analysis (Dupont and Plummer, 1990). All statistical analyses were performed in additive (comparison of wild type homozygotes, heterozygotes and polymorphic homozygotes) and dominant genetic model (comparison of wild type homozygotes with heterozygotes and polymorphic homozygotes combined; marked as Pdom), using IBM SPSS Statistics 27 (IBM Corporation, NY, United States).

## 3 Results

### 3.1 Demographic and clinical characteristics of patients with COVID-19

Of the 107 patients with COVID-19 who qualified for the present study, 2 had moderate (1.9%), 88 severe (83%) and 16 critical (15.1%) disease (data is missing for one patient). Since only two patients had moderate disease, we excluded them from further statistical analysis. Thus, the assessment of the association of genetic polymorphisms in glucocorticoid pathway with the course and short-term outcomes of COVID-19 was performed in 105 patients. Their median (25%–75%) age was 62 (53–71) years, 74 (70.5%) were males and 31 (29.5%) were females. Demographic and clinical characteristics of patients with COVID-19 are presented in Table 1.

**TABLE 1:** Demographic and clinical characteristics of the patients with COVID-19.

Characteristic (total N = 105)		
Gender	Male, n (%)	74 (70.5)
	Female, n (%)	31 (29.5)
Age	Years, median (25%–75%)	62 (53–71)
BMI^a^	Median (25%–75%)	31.9 (27.7–35.9)
Chronic disease	Total, n (%)	77 (73.3)
	Diabetes, n (%)	22 (28.6)
	Hyperlipidemia, n (%)	26 (33.8)
	Arterial hypertension, n (%)	52 (67.5)
	Heart failure, n (%)	6 (7.8)
	Chronic lung disease, n (%)	13 (16.9)
Duration of dexamethasone treatment^b^	Median, days (25%–75%)	5.5 (4–8)
Switching to methylprednisolone treatment	Yes, n (%)	27 (25.7)
	No, n (%)	78 (74.3)
COVID-19 severity^b^	Severe, n (%)	88 (84.6)
	Critical, n (%)	16 (15.4)
Short-term outcomes	Duration of hospitalization (days)^b^, median (25%–75%)	9.5 (7–14.75)
	Duration of oxygen supplementation (days)^c^, median (25%–75%)	7 (5–11)
	Admission to ICU, Yes, n (%)	12 (11.4)
	Admission to ICU, No, n (%)	93 (88.6)

^a^ Data missing for 33 patients.

^b^ Data missing for one patient.

^c^ Data missing for 3 patients.

### 3.2 Genetic polymorphisms analysis

All genotype distributions were in accordance with Hardy-Weinberg equilibrium (HWE), except for *CYP3A5* rs10264272 as all the patients were homozygous for the polymorphic allele (Supplementary Table S1). For this reason, we have excluded *CYP3A5* rs10264272 from further analysis.

### 3.3 Association of glucocorticoid pathway polymorphisms with COVID-19 severity

None of the evaluated clinical parameters (presence of underlying disease, age, gender and BMI) were associated with disease severity (all *p* > 0.05). We observed higher odds (OR = 6.538; 95% CI = 1.19–35.914: *p* = 0.031) for critical COVID-19 disease in carriers of *CYP3A4* rs35599367 polymorphic T allele. None of the other investigated polymorphisms showed associations with COVID-19 severity (Table 2).

**TABLE 2:** The associations of glucocorticoid pathway polymorphisms with COVID-19 severity.

		N (%)	N (%)	OR	95% CI	*p*-value
Polymorphisms	Genotype	Critical N = 16	Severe N = 87			
*NR3C1* rs6198	TT	11 (68.8)	64 (73.6)	Ref.		
	TC	3 (18.8)	22 (25.3)	0.793	0.203–3.108	0.740
	CC	2 (12.5)	1 (1.1)	11.636	0.97–139.543	0.053
	TC + CC	5 (31.3)	23 (26.4)	1.265	0.397–4.033	0.691
*NR3C1* rs33388	AA	7 (53.8)	21 (23.9)	Ref.		
	AT	6 (37.5)	46 (52.3)	0.391	0.117–1.308	0.127
	TT	3 (18.5)	21 (23.9)	0.429	0.097–1.886	0.262
	AT + TT	9 (56.3)	67 (76.1)	0.403	0.134–1.214	0.106
*NR3C1* rs33389	CC	3 (18.8)	21 (23.9)	Ref.		
	CT	6 (37.5)	46 (52.3)	0.913	0.208–4.007	0.904
	TT	7 (43.8)	21 (23.9)	2.333	0.53–10.267	0.262
	CT + TT	13 (81.3)	67 (76.1)	1.358	0.353–5.227	0.656
*ABCB1* rs1045642	TT	4 (25)	23 (26.1)	Ref.		
	CT	6 (37.5)	51 (58)	0.676	0.174–2.629	0.573
	CC	6 (37.5)	14 (15.9)	2.464	0.59–10.287	0.216
	CT + CC	12 (75)	65 (73.9)	1.062	0.311–3.622	0.924
*ABCB1* rs1128503	TT	4 (25)	14 (15.9)	Ref.		
	CT	4 (25)	45 (51.1)	0.311	0.069–1.408	0.130
	CC	8 (50)	29 (33)	0.966	0.248–3.759	0.960
	CT + CC	12 (75)	74 (54.1)	0.568	0.16–2.016	0.381
*ABCB1* rs2032582	GG	7 (53.8)	26 (34.7)	Ref.		
	GT	2 (15.4)	36 (48)	0.206	0.040–1.075	0.061
	TT + TA	4 (30.8)	13 (17.3)	1.143	0.283–4.622	0.851
	TT + TA + GT	6 (46.2)	49 (65.3)	0.455	0.138–1.494	0.194
*GSTP1* rs1695	TT	3 (18.8)	15 (17)	Ref.		
	TC	4 (25)	44 (50)	0.455	0.091–2.268	0.336
	CC	9 (56.3)	29 (33)	1.552	0.365–6.600	0.552
	TC + CC	13 (81.3)	73 (86)	0.89	0.226–3.514	0.868
*GSTP1* rs1138272	CC	13 (81.3)	73 (83)	Ref.		
	CT	3 (18.8)	14 (15.9)	1.203	0.303–4.781	0.793
	TT	0 (0)	1 (1.1)	0	—	1
	CT + TT	3 (18.8)	15 (17)	1.123	0.285–4.432	0.838
*GSTM1*	No deletion	7 (43.8)	42 (47.7)	Ref.		
	Gene deletion	9 (56.3)	46 (52.3)	1.174	0.402–3.431	0.770
*GSTT1*	No deletion	14 (87.5)	67 (76.1)	Ref.		
	Gene deletion	2 (12.5)	21 (23.9)	0.456	0.096–2.17	0.324
*CYP3A4* rs35599367	CC	13 (81.3)	85 (96.6)	Ref.		
	TC	3 (18.8)	3 (3.4)	**6.538**	**1.19–35.914**	**0.031**
*CYP3A4* rs2740574	AA	15 (93.8)	85 (86.6)	Ref.		
	GA	1 (6.3)	3 (3.4)	1.889	0.184–19.390	0.592
*CYP3A5* rs776746	CC	12 (75)	76 (87.4)	Ref.		
	TC	4 (25)	10 (11.5)	2.533	0.684–9.386	0.164
	TT	0 (0)	1 (1.1)	0	—	1
	TC + TT	4 (25)	11 (12.6)	2.303	0.63–8.419	0.207

Statistically and nominally significant associations are printed in bold.

### 3.4 The associations of glucocorticoid pathway polymorphisms with the need for ICU treatment

None of the selected clinical parameters (presence of chronic disease, age, gender, and BMI) showed association with the need for ICU treatment (all *p* > 0.05). Carriers of one *NR3C1* rs33388 polymorphic allele had lower odds for treatment in ICU (OR = 0.144; 95% CI = 0.027–0.769: *p* = 0.023) compared to non-carriers. On the contrary, carriers of *CYP3A4* rs35599367 polymorphic allele (OR = 10; 95% CI = 1.754–57.021: *p* = 0.01), as well as carriers of one *GSTP1* rs1138272 polymorphic allele (OR = 4.762; 95% CI = 1.3–17.441: *p* = 0.018) had higher odds for treatment in ICU (Table 3).

**TABLE 3:** Association of glucocorticoid pathway polymorphisms with the need for ICU treatment.

		N (%)	N (%)	OR	95% CI	*p*-value
Polymorphism	Genotype	ICU treatment N = 12	No ICU treatment N = 92			
*NR3C1* rs6198	TT	11 (91.7)	65 (70.7)		Ref.	
	TC	1 (8.3)	24 (26.1)	0.246	0.030–2.011	0.191
	CC	0 (0)	3 (3.3)	0	—	0.999
	TC + CC	1 (8.3)	27 (29.3)	0.219	0.027–1.78	0.155
***NR3C1* rs33388**	AA	6 (50)	22 (23.7)		Ref.	
	AT	2 (16.7)	51 (54.8)	**0.144**	**0.027–0.769**	**0.023**
	TT	4 (33.3)	20 (21.5)	0.733	0.18–2.982	0.665
	AT + TT	6 (50)	71 (76.3)	0.31	0.091–1.059	0.062
*NR3C1* rs33389	CC	4 (33.3)	20 (21.5)		Ref.	
	CT	2 (16.7)	51 (54.8)	0.196	0.033–1.156	0.072
	TT	6 (50)	22 (23.7)	1.364	0.335–5.544	0.665
	CT + TT	8 (66.7)	73 (78.5)	0.548	0.15–2.007	0.364
*ABCB1* rs1045642	TT	3 (25)	24 (25.8)		Ref.	
	CT	6 (50)	51 (54.8)	0.941	0.217–4.087	0.936
	CC	3 (25)	18 (19.4)	1.333	0.24–7.394	0.742
	CT + CC	9 (75)	69 (74.2)	1.043	0.261–4.176	0.952
*ABCB1* rs1128503	TT	3 (25)	15 (16.1)		Ref.	
	CT	3 (25)	47 (50)	0.319	0.058–1.752	0.189
	CC	6 (50)	31 (33.3)	0.968	0.212–4.411	0.966
	CT + CC	9 (75)	78 (83.9)	0.577	0.14–2.384	0.447
*ABCB1* rs2032582	GG	4 (40)	30 (38)		Ref.	
	GT	2 (20)	36 (45.6)	0.417	0.071–2.434	0.331
	TT + TA	4 (40)	13 (16.5)	2.308	0.499–10.669	0.284
	TT + TA + GT	6 (60)	49 (62)	0.918	0.239–3.522	0.901
*GSTP1* rs1695	AA	3 (25)	15 (16.)		Ref.	
	AG	3 (25)	46 (49.5)	0.326	0.059–1.791	0.197
	GG	6 (50)	32 (34.4)	0.937	0.206–4.267	0.933
	AG + GG	9 (75)	78 (83.9)	0.577	0.14–2.384	0.447
*GSTP1* rs1138272	CC	7 (58.3)	80 (86)		Ref.	
	CT	5 (41.7)	12 (12.9)	**4.762**	**1.3–17.441**	**0.018**
	TT	0 (0)	1 (1.1)	0	—	1
	CT + TT	5 (41.7)	13 (14)	4.396	1.212–15.947	0.062
*GSTM1*	No deletion	5 (41.7)	45 (48.4)		Ref.	
	Gene deletion	7 (58.3)	48 (51.6)	1.312	0.388–4.435	0.662
*GSTT1*	No deletion	10 (83.3)	71 (76.3)		Ref.	
	Gene deletion	2 (16.7)	22 (23.7)	0.645	0.131–3.171	0.590
*CYP3A4* rs35599367	CC	9 (75)	90 (96.8)		Ref.	
	TC	3 (25)	3 (3.2)	**10**	**1.754–57.021**	**0.010**
*CYP3A4* rs2740574	AA	11 (91.7)	90 (96.8)		Ref.	
	GA	1 (8.3)	3 (3.2)	2.727	0.261–28.544	0.402
*CYP3A5* rs776746	CC	9 (75)	80 (87)		Ref.	
	CT	3 (25)	11 (12)	2.424	0.568–10.342	0.232
	TT	0 (0)	1 (1.1)	0	—	1
	CT + TT	3 (25)	12 (13)	2.222	0.526–9.385	0.277

Statistically and nominally significant associations are printed in bold.

### 3.5 Association of glucocorticoid pathway polymorphisms with duration of hospitalization and duration of oxygen supplementation

Chronic diseases were associated with the median duration of hospitalization (*p* = 0.009) and for that reason all the genetic association models were adjusted for chronic diseases. The median duration of hospitalization was nominally shorter in carriers of one polymorphic *ABCB1* rs1128503 (8 days) when compared to homozygotes for reference or polymorphic alleles (both 12 days) (*p* = 0.028), as well as in *GSTP1* rs1695 heterozygotes (8 days) compared to homozygotes for *GSTP1* rs1695 reference or polymorphic alleles (both 12 days) (*p* = 0.015). None of these associations retained significance after adjustment for chronic disease. On the other hand, associations between *NR3C1* rs6198 (*p*_*adj*_ = 0.001), *NR3C1* rs33388 (*p*_*adj*_ = 0.025, *Pdom*_*adj*_ = 0.006), *NR3C1* rs33389 (*p*_*adj*_ = 0.025), and *CYP3A4* rs35599367 (*p*_*adj*_ = 0.022) with duration of hospitalization became significant after the adjustments for chronic diseases. Homozygotes for polymorphic *NR3C1* rs6198 allele needed significantly longer hospitalization (median 45 days) compared to heterozygotes (11 days) or non-polymorphic homozygotes (9 days) (*p*_*adj*_ = 0.001). Homozygotes for polymorphic *NR3C1* rs33389 allele also needed longer (12 days) hospitalization when compared to carriers of one or two reference alleles (both 9 days) (*p*_*adj*_ = 0.025). On the other hand, the median duration of hospitalization was longer in homozygotes *for NR3C1* rs33388 reference allele (12 days) when compared to carriers of one or two polymorphic alleles (both 9 days) (*Pdom*_*adj*_ = 0.006). The median duration of hospitalization in the carriers of *CYP3A4* rs35599367TC genotype was 16 days longer when compared to the carriers of the CC genotype (*p*_*adj*_ = 0.022) (Table 4).

**TABLE 4:** Association of glucocorticoid pathway polymorphisms with duration of hospitalization and duration of oxygen supplementation.

		Duration of hospitalization			Duration of oxygen supplementation		
Polymorphism	Genotype	Median (25–75%)	*p*-value	*p*-value adj^a^	Median (25–75%)	*p*-value	*p*-value adj^b^
***NR3C1* rs6198**	TT	9 (7–11)	0.259	**0.001**	7 (5–10, 25)	0.227	**0.001**
	TC	11 (7–15.5)			8 (5–11)		
	CC	45 (26.5–45.5)^c^			39 (5–/)^d^		
	TC + CC	11 (7.25–16.75)	Pdom = 0.518	Pdom = 0.294	8 (5–13)	Pdom = 0.234	Pdom = 0.134
***NR3C1* rs33388**	AA	12 (8–20)	0.120	**0.025**	8 (5–16)	0.491	0.074
	AT	9 (7–13.75)			8 (5–10)		
	TT	9 (7–13.75)			6 (5–9)		
	AT + TT	9 (7–13.75)	Pdom = 0.040	**Pdom = 0.006**	7 (5–10)	Pdom = 0.288	**Pdom = 0.022**
***NR3C1* rs33389**	CC	9 (7–13.75)	0.120	**0.025**	6 (5–9)	0.491	0.074
	CT	9 (7–13.75)			8 (5–10)		
	TT	12 (8–20)			8 (5–16)		
	CT + TT	11 (7–15)	Pdom = 0.441	Pdom = 0.362	8 (5–11)	Pdom = 0.392	Pdom = 0.432
***ABCB1* rs1045642**	TT	9.5 (7–15.5)	0.306	0.206	8 (5–10.5)	0.321	0.354
	CT	9 (7–13)			7 (5–10)		
	CC	12 (8.5–19)			10 (5–17)		
	CT + CC	9.5 (7–13.25)	Pdom = 0.925	Pdom = 0.459	7 (5–11)	Pdom = 0.842	Pdom = 0.794
***ABCB1* rs1128503**	TT	12 (7.5–16.5)	**0.028**	0.183	8 (5–12)	0.060	0.222
	CT	8 (7–12.25)			6 (4–9)		
	CC	12 (8–16)			8.5 (5.25–15.5)		
	CT + CC	9 (7–14)	Pdom = 0.373	Pdom = 0.364	7 (5–11)	Pdom = 0.808	Pdom = 0.780
***ABCB1* rs2032582**	GG	11.5 (8–16)	0.133	0.086	8 (5.5–14.5)	0.107	0.111
	GT	9 (7–12.5)			6 (4–9)		
	TT + TA	12 (6.5–18)			7.5 (4.25–11)		
	TT + TA + GT	9 (7–13.25)	Pdom = 0.072	Pdom = 0.150	7 (4–9.25)	**Pdom = 0.047**	Pdom = 0.074
***GSTP1* rs1695**	AA	12 (7.5–17)	**0.015**	0.133	8 (5–11)	**0.047**	0.215
	AG	8 (7–12)			6 (4–9)		
	GG	12 (8.75–16)			9 (5.5–15)		
	AG + GG	9 (1–14)	Pdom = 0.359	Pdom = 0.283	7 (5–11)	Pdom = 0.971	Pdom = 0.842
***GSTP1* rs1138272**	CC	9 (7–15)	0.759	0.961	7 (5–11)	0.753	0.881
	CT	11 (7.5–14.5)			7 (4–12.5)		
	TT	12^e^			10^e^		
	CT + TT	11.5 (7.75–14.25)	Pdom = 0.490	Pdom = 0.890	8.5 (4–11.75)	Pdom = 0.625	Pdom = 0.658
***GSTM1***	No deletion	9 (7–15.5)	0.938	0.373	7,5 (5–11)	0.642	0.414
	Gene deletion	11 (7–14)			7 (4.75–11)		
***GSTT1***	No deletion	11 (7–15.75)	0.178	0.206	7 (5–11)	0.754	0.402
	Gene deletion	9 (7–11.75)			7 (5–11)		
***CYP3A4* rs35599367**	CC	9 (7–14)	0.069	**0.022**	7 (5–11)	0.064	**0.040**
	TC	25 (10.5–34)			14.5 (7.5–25.75)		
***CYP3A4* rs2740574**	AA	9.5 (7–14.75)	0.678	0.083	7 (5–11)	0.835	0.206
	GA	10.5 (7.25–43.75)			6.5 (5.25–28.75)		
***CYP3A5* rs776746**	CC	9 (7–14)	0.341	0.341	7 (5–11)	0.473	0.564
	CT	11.5 (7.75–20.5)			8 (5–16)		
	TT	13^e^			10^e^		
	CT + TT	12 (8–19)	Pdom = 0.153	Pdom = 0.162	9 (5–15)	Pdom = 0.247	Pdom = 0.316

Statistically and nominally significant associations are printed in bold.

^a^ Adjusted for chronic diseases.

^b^ Adjusted for chronic diseases and age.

^c^ Determined by Tukey’s hinges.

^e^ One patient in the group.

^d^ Due to the low representation of patients with the CC, genotype, we could not statistically accurately calculate the 75% percentile.

Age (*p* = 0.048) was associated with duration of oxygen supplementation, while chronic diseases (*p* = 0.053) showed a tendency for association, but both variables were adjusted for in this part of the multivariable analysis. With regards to *GSTP1* rs1695 polymorphism the median duration of oxygen supplementation was the longest in homozygotes for the polymorphic allele (9 days) (*p* = 0.047). The nominal association between *ABCB1* rs2032582 polymorphism and duration of oxygen supplementation observed in the dominant genetic model (*Pdom* = 0.047), was lost after the adjustment for the age and chronic diseases. On the other hand, the associations between *NR3C1* rs6198 (*p*_*adj*_ = 0.001), *NR3C1* rs33388 (*Pdom*_*adj*_ = 0.022), *CYP3A4* rs35599367 (*p*_*adj*_ = 0.040) and duration of oxygen supplementation became significant or nominally significant after the adjustments. Homozygotes for polymorphic *NR3C1* rs6198 allele needed significantly longer oxygen supplementation (median 39 days) compared to heterozygotes (8 days) or homozygotes for the reference allele (7 days) (*p*_*adj*_ = 0.001). Carriers of one or two *NR3C1* rs33388 polymorphic allele had only slightly shorter median duration of oxygen supplementation (7 days), compared to non-carriers (8 days) (*Pdom* = 0.022). The median duration of oxygen supplementation in *CYP3A4* rs35599367TC genotype carriers was 7.5 days longer when compared to CC genotype carriers (*p*_*adj*_ = 0.040) (Table 4).

### 3.6 Association of glucocorticoid pathway polymorphisms with duration of dexamethasone treatment

None of the selected clinical parameters (presence of chronic diseases, age, gender and BMI) showed association with duration of dexamethasone treatment (all *p* > 0.05). Among the investigated polymorphisms, three were associated with duration of dexamethasone treatment. The median duration of dexamethasone treatment was nominally shorter in carriers of two polymorphic *NR3C1* rs6198 alleles, compared to non-carriers (*p* = 0.043, *Pdom* = 0.014). *ABCB1* rs1128503 and *GSTP1* rs1695 polymorphism were also associated with nominally shorter dexamethasone treatment (*p* = 0.025, *Pdom* = 0.022; and *p* = 0.022, *Pdom* = 0.031, respectively) (Table 5).

**TABLE 5:** Association of glucocorticoid pathway polymorphisms with duration of dexamethasone treatment.

		Duration	
Polymorphism	Genotype	Median (25–75%)	*p*-value
*NR3C1* rs6198	TT	6 (4–8.5)	**0.043**
	TC	4 (2–7)	
	CC	3 (3–/)^a^	
	TC + CC	4 (2.25–6.75)	**Pdom = 0.014**
***NR3C1* rs33388**	AA	6 (3–8)	0.567
	AT	6 (4–9)	
	TT	5 (3.75–7.25)	
	AT + TT	5 (4–8)	Pdom = 0.436
***NR3C1* rs33389**	CC	5 (3.75–7.25)	0.567
	CT	6 (4–9)	
	TT	6 (3–8)	
	CT + TT	6 (4–8.75)	Pdom = 0.664
***ABCB1* rs1045642**	TT	6 (4–10)	0.060
	CT	5.5 (4–8)	
	CC	4 (2–8)	
	CT + CC	5 (3–8)	Pdom = 0.055
*ABCB1* rs1128503	TT	7 (5–10.5)	**0.025**
	CT	6 (4–8)	
	CC	5 (2–7)	
	CT + CC	5 (3–8)	Pdom = **0.022**
***ABCB1* rs2032582**	GG	5 (3–8.75)	0.406
	GT	6 (4–8)	
	TT + TA	6 (4–9)	
	TT + TA + GT	6 (4–8)	Pdom = 0.180
*GSTP1* rs1695	AA	7 (5–10.5)	**0.022**
	AG	6 (4–8)	
	GG	5 (2.25–7)	
	AG + GG	5 (3–8)	Pdom = **0.031**
***GSTP1* rs1138272**	CC	5 (3–8)	0.379
	CT	5 (3.25–10)	
	TT	10^b^	
	CT + TT	6 (3.5–10)	Pdom = 0.604
***GSTM1***	No deletion	6 (4–9.5)	0.347
	Gene deletion	5 (3–8)	
***GSTT1***	No deletion	5 (3–8)	0.475
	Gene deletion	6 (4–8)	
***CYP3A4* rs35599367**	CC	6 (4–8)	0.424
	TC	4 (2.5–8.5)	
***CYP3A4* rs2740574**	AA	5.5 (4–8)	0.714
	GA	5.5 (2–7.5)	
***CYP3A5* rs776746**	CC	5 (4–8)	0.480
	TC	6 (3.75–9.25)	
	TT	9^b^	
	CT + TT	6 (4–9)	Pdom = 0.416

Statistically and nominally significant associations are printed in bold.

^a^ Due to the low representation of patients with the CC, genotype, we could not statistically accurately calculate the 75% percentile.

^b^ One patient in group.

### 3.7 Association of glucocorticoid pathway polymorphisms with switching from dexamethasone to methylprednisolone treatment

As chronic diseases showed association with odds for switching from dexamethasone to methylprednisolone treatment (*p* = 0.043), genetic model was adjusted for chronic diseases. Carriers of one polymorphic *NR3C1* rs6198 allele had higher odds for switching from dexamethasone to methylprednisolone treatment (OR = 2.711; 95% CI = 1.018–7.22: *p* = 0.046) compared to non-carriers. This association remained significant after adjustment for chronic diseases (OR = 3.363; 95% CI = 1.185–9.545: *p* = 0.023). On the contrary, carriers of polymorphic *NR3C1* rs33388 allele had lower odds for switching the treatment (OR = 0.359; 95% CI = 0.129–1.001: *p* = 0.05) compared to non-carriers, but this association was no longer significant after adjustment (OR = 0.363; 95% CI = 0.128–1.035: *p* = 0.058). *ABCB1* rs2032582 polymorphism was also associated with lower odds for treatment switching (OR = 0.245; 95% CI = 0.076–0.787: *p* = 0.018), this association remaining significant after adjustment. *CYP3A4* rs35599367 polymorphism was associated with higher odds for treatment switching (OR = 6.609; 95% CI = 1.137–38.424: *p* = 0.036), but this association did not remain significant after the adjustment for chronic diseases (Table 6).

**TABLE 6:** Association of glucocorticoid pathway polymorphisms with switching from dexamethasone to methylprednisolone treatment.

		N (%)	N (%)	OR	95% CI	*p*-value	OR adj^a^	95% CI adj^a^	*p*-value adj^a^
Polymorphism	Genotype	Treatment switched N = 27	No treatment switching N = 77						
*NR3C1* rs6198	TT	15 (55.6)	61 (79.2)	Ref.			Ref.		
	TC	10 (37)	15 (19.5)	**2.711**	**1.018–7.22**	**0.046**	**3.363**	**1.185–9.545**	**0.023**
	CC	2 (7.4)	1 (1.3)	8.133	0.691–95.774	0.096	6.298	0.532–74.63	0.145
	TC + CC	12 (44.4)	16 (20.8)	**3.05**	**1.194–7.79**	**0.020**	**3.641**	**1.347–9.841**	**0.011**
*NR3C1* rs33388	AA	11 (40.7)	17 (21.8)	Ref.			Ref.		
	AT	10 (37)	43 (55.1)	**0.359**	**0.129–1.001**	**0.050**	0.363	0.128–1.035	0.058
	TT	6 (22.2)	18 (23.1)	0.515	0.156–1.702	0.277	0.571	0.167–1.952	0.372
	AT + TT	16 (59.3)	61 (78.2)	0.405	0.159–1.035	0.059	0.421	0.161–1.099	0.077
***NR3C1* rs33389**	CC	6 (22.2)	18 (23.1)	Ref.			Ref.		
	CT	10 (37)	43 (55.1)	0.698	0.22–2.208	0.540	0.636	0.195–2.073	0.453
	TT	11 (40.7)	17 (21.8)	1.941	0.587–6.415	0.277	1.751	0.512–5.982	0.372
	CT + TT	21 (77.8)	60 (76.9)	1.05	0.368–2.998	0.927	0.954	0.324–2.804	0.931
***ABCB1* rs1045642**	TT	5 (18.5)	22 (28.2)	Ref.			Ref.		
	CT	13 (48.1)	44 (56.4)	1.3	0.411–4.111	0.655	1.082	0.331–3.536	0.896
	CC	9 (33.3)	12 (15.4)	3.3	0.899–12.108	0.072	2.738	0.72–10.412	0.139
	CT + CC	22 (81.5)	56 (71.8)	1.729	0.582–5.137	0.325	1.437	0.468–4.411	0.526
***ABCB1* rs1128503**	TT	4 (14.8)	14 (17.9)	Ref.			Ref.		
	CT	8 (29.6)	42 (53.8)	0.667	0.174–2.556	0.554	0.635	0.161–2.499	0.516
	CC	15 (55.6)	22 (28.2)	2.386	0.657–8.674	0.187	2.121	0.566–7.943	0.264
	CT + CC	23 (85.2)	64 (82.1)	1.258	0.375–4.214	0.710	1.161	0.336–4.007	0.813
*ABCB1* rs2032582	GG	13 (59.1)	21 (31.3)	Ref.			Ref.		
	GT	5 (22.7)	33 (49.3)	**0.245**	**0.076–0.787**	**0.018**	**0.222**	**0.067–0.734**	**0.014**
	TT + TA	4 (18.2)	13 (19.4)	0.497	0.133–1.855	0.298	0.606	0.152–2.406	0.476
	TT + TA + GT	9 (40.9)	46 (68.7)	**0.316**	**0.117–0.854**	**0.023**	**0.311**	**0.112–0.866**	**0.025**
***GSTP1* rs1695**	AA	4 (14.8)	14 (17.9)	Ref.			Ref.		
	AG	7 (25.9)	42 (53.8)	0.583	0.148–2.294	0.440	0.513	0.126–2.086	0.351
	GG	16 (59.3)	22 (28.2)	2.545	0.705–9.195	0.154	2.12	0.565–7.953	0.265
	AG + GG	23 (85.2)	64 (82.1)	1.258	0.375–4.214	0.710	1.074	0.309–3.737	0.911
***GSTP1* rs1138272**	CC	23 (85.2)	64 (82.1)	Ref.			Ref.		
	CT	4 (14.8)	13 (16.7)	0.856	0.253–2.894	0.803	0.89	0.256–3.086	0.854
	TT	0 (0)	1 (1.3)	0	—	1	0	—	1
	CT + TT	4 (14.8)	14 (17.9)	0.795	0.237–2.664	0.710	0.861	0.25–2.972	0.813
***GSTM1***	No deletion	13 (48.1)	37 (47.4)	Ref.			Ref.		
	Gene deletion	14 (51.9)	41 (52.6)	0.972	0.405–2.334	0.949	0.943	0.385–2.311	0.898
***GSTT1***	No deletion	23 (85.2)	58 (74.4)	Ref.			Ref.		
	Gene deletion	4 (14.8)	20 (25.6)	0.504	0.155–1.637	0.254	0.48	0.145–1.587	0.229
*CYP3A4* rs35599367	CC	23 (85.2)	76 (97.4)	Ref.			Ref.		
	TC	4 (14.8)	2 (2.6)	**6.609**	**1.137–38.424**	**0.036**	5.1	0.865–30.073	0.072
***CYP3A4* rs2740574**	AA	26 (96.3)	75 (96.2)	Ref.			Ref.		
	GA	1 (3.7)	3 (3.8)	0.962	0.096–9.655	0.973	0.942	0.09–9.908	0.960
***CYP3A5* rs776746**	CC	22 (84.6)	67 (85.9)	Ref.			Ref.		
	CT	4 (15.4)	10 (12.8)	1.218	0.347–4.276	0.758	1.027	0.284–3.712	
	TT	0 (0)	1 (1.3)	0	—	1	0	—	1
	CT + TT	4 (15.4)	11 (14.1)	1.107	0.32–3.833	0.872	0.921	0.259–3.272	0.898

Statistically and nominally significant associations are printed in bold.

^a^ Adjusted for chronic diseases.

### 3.8 Association of haplotypes with COVID-19 severity and treatment outcomes

Only haplotypes with the frequency above 5% were included in the analysis, with the exception of *CYP3A4*, due to low frequency of polymorphic *CYP3A4* rs35599367 and rs2740574 alleles in our study group. In haplotype analysis all four GCs pathway genes showed nominally or statistically significant associations with at least one studied outcome. *NR3C1* rs6198-rs33388-rs33389 CAT haplotype was associated with longer hospitalization (*p* = <0.001), longer duration of oxygen supplementation (*p* = <0.001) and higher odds for switching from dexamethasone to methylprednisolone when compared to TTC haplotype (OR = 2.991; 95% CI = 1.251–7.148: *p* = 0.013). When compared to the most common haplotype, *ABCB1* rs1045642-rs1128503-rs2032582 TTT haplotype as well as *GSTP1* rs1695-rs1138272 CC haplotype carriers had lower odds for switching from dexamethasone to methylprednisolone (OR = 0.328; 95% CI = 0.154–0.701: *p* = 0.004 and OR = 0.465; 95% CI = 0.238–0.910: *p* = 0.026, respectively). Furthermore, *GSTP1* CC haplotype was also associated with longer dexamethasone treatment (*p* = 0.009). Regarding *CYP3A4* rs35599367-rs2740574 haplotypes, TA haplotype carriers had higher odds for more severe COVID-19 (OR = 6.833; 95% CI = 1.234–37.825: *p* = 0.028) and for the need for ICU treatment (OR = 10.875; 95% CI = 1.877–62.992: *p* = 0.008), when compared to CA haplotype. TA haplotype was also associated with longer hospitalization (*p* = <0.001), longer duration of oxygen supplementation (*p* = <0.001) and switching to methylprednisolone (OR = 6.636; 95% CI = 1.138–38.692: *p* = 0.035), while CG was associated with longer hospitalization (*p* = <0.001), when compared with the most common *CYP3A4* CA haplotype (Table 7).

**TABLE 7:** Association of haplotypes with COVID-19 severity and treatment outcomes.

		COVID-19 severity		EIT		Duration of hospitalization		Duration of oxygen supplementation		Duration of dexamethasone treatment		Switching dexamethasone to methylprednisolone	
Gene	Haplotype (%)	Or (95%CI)	*p*-value	Or (95%CI)	*p*-value	Diff. (95%CI)	*p*-value	Diff. (95%CI)	*p*-value	Diff. (95%CI)	*p*-value	Or (95%CI)	*p*-value
*NR3C1*	TTC (0.48)	Ref.		Ref.		Ref.		Ref.		Ref.		Ref.	
	TAT (0.37)	1.517 (0.698–3.294)	0.293	1.742 (0.829–3.663)	0.143	2.42 (−0.27–5.11)	0.078	1.60 (−1.04–4.24)	0.234	0.41 (−0.44–1.26)	0.343	1.136 (0.585–2.205)	0.706
	CAT (0.15)	2.160 (0.807–5.783)	0.125	0.322 (0.038–2.701)	0.296	4.77 (1.95–7.58)	<0.001	5.23 (2.74–7.73)	<0.001	−1.17 (−2.44–0.10)	0.071	2.991 (1.251–7.148)	0.013
*ABCB1*	CCG (0.40)	Ref.		Ref.		Ref.		Ref.		Ref.		Ref.	
	TTT (0.34)	0.578 (0.269–1.240)	0.159	1.154 (0.481–2.769)	0.748	−2.21 (−4.56–0.13)	0.065	−2.75 (−4.80–−0.70)	0.009	0.91 (−0.05–1.88)	0.063	0.328 (0.154–0.701)	0.004
	CTG (0.14)	0.409 (0.084–1.979)	0.266	0.846 (0.160–4.464)	0.844	−1.83 (−7.41–3.75)	0.520	−2.31 (−7.52–2.89)	0.384	0.41 (−0.90–1.71)	0.543	0.716 (0.224–2.289)	0.573
*GSTP1*	TC (0.54)	Ref.		Ref.		Ref.		Ref.		Ref.		Ref.	
	CC (0.37)	0.615 (0.277–1.367)	0.233	0.973 (0.387–2.450)	0.954	−1.25 (−4.05–1.54)	0.379	−2.06 (−4.98–0.87)	0.167	1.12 (0.28–1.97)	0.009	0.465 (0.238–0.910)	0.026
	TT (0.06)	0.938 (0.167–5.266)	0.942	3.621 (0.920–14.249)	0.066	−1.24 (−15.29–12.80)	0.862	−1.01 (−9.81–7.79)	0.823	0.83 (−0.79–2.44)	0.316	0.716 (0.157–3.279)	0.667
*CYP3A4*	CA (0.95)	Ref.		Ref.		Ref.		Ref.		Ref.		Ref.	
	TA (0.03)	6.833 (1.234–37.825)	0.028	10.875 (1.877–62.992)	0.008	10.86 (4.86–16.87)	<0.001	4.49 (−0.43–9.41)	<0.001	−0.89 (−3.13–1.35)	0.434	6.636 (1.138–38.692)	0.035
	CG (0.02)	2.278 (0.219–23.714)	0.491	3.625 (0.337–39.021)	0.288	8.56 (4.25–12.87)	<0.001	7.99 (3.46–12.51)	0.074	−0.89 (−4.12–2.33)	0.587	1.106 (0.109–11.175)	0.932

Statistically and nominally significant associations are printed in bold. Diff—Mean difference.

## 4 Discussion

The primary objective of this study was to investigate the associations between common genetic polymorphisms within the glucocorticoid pathway and treatment outcomes in hospitalized patients with COVID-19. Our findings bring novel evidence of *NR3C1* rs6198, *GSTP*1 rs1695, and *CYP3A4* rs35599367 polymorphisms having impact on COVID-19 severity, the course of the disease, and the outcome of treatment with dexamethasone and methylprednisolone. To our knowledge, this is the first known experimental study using pathway based approach to investigate common functional polymorphisms in glucocorticoid metabolic pathway to find promising genetics biomarkers for predicting the outcome of patients with COVID-19 treated with GCs.

First, we observed strong associations of *CYP3A4* rs35599367 with several studied outcomes. *CYP3A4* rs35599367 polymorphism is located in intron 6 and causes decreased mRNA expression, leading to lower enzyme activity. This may results in increased drug exposure and adverse drug reactions (ADRs) occurrence (Wang and Sadee, 2016). In our study, carriers of one *CYP3A4* rs35599367 polymorphic allele had six-times higher odds for critical COVID-19 disease and higher odds for ICU treatment when compared to reference homozygotes. This polymorphism was also associated with worse treatment outcomes, namely, with longer hospitalization, longer requirement for oxygen supplementation and higher odds for switching the treatment to methylprednisolone. These findings suggest that this polymorphism could be a genetic marker for prediction of a more severe course of COVID-19 and less favorable treatment outcomes. In previous studies, *CYP3A4* rs35599367 was associated with improved asthma control with glucocorticoid treatment. Despite the prevalent expression of CYP3A5 and CYP3A7 in human lung tissue, reduced activity of pulmonary CYP3A4 enzyme was suggested to prolong the presence of active glucocorticoid within the airways, enhancing its efficacy (Stockmann et al., 2013). Decreased enzyme activity due to *CYP3A4* rs35599367 was also associated with concentration-dependent toxicity caused by cyclosporine in recipients of kidney transplants (Elens et al., 2012), as well as a lower tacrolimus dose requirement in kidney transplant recipients (Elens et al., 2011), further emphasizing the important role of *CYP3A4* in immunosuppression.

Regarding *GSTP1*, both rs1138272 (*p*.Ala114Val) and rs1695 (*p*.Ile105Val) polymorphisms showed important associations with the studied outcomes. Carriers of one *GSTP1* rs1138272 polymorphic allele were more likely to need ICU treatment, but this polymorphism did not show significant associations with disease severity or any other studied outcomes. On the other hand, *GSTP1* rs1695 was not associated with the odds for ICU treatment, but it was associated with duration of hospitalization, oxygen supplementation and dexamethasone treatment. The minor *GSTP*1 rs1695 G allele has been previously associated with lower efficiency of GCs conjugation in phase 2 of the biotransformation. The active site of GSTP1 enzyme is composed from G-site and H-site. The p. 105 Ile > Val amino acid substitution is located in the H-site, and causes decreased enzyme stability (Johansson et al., 1998), resulting in lower GSTP1 conjugation capacity (Gasic et al., 2018). The decreased GSTP1 activity was reported to result in prolonged glucocorticoid exposure and the occurrence of ADRs (Gasic et al., 2018). These reports could explain our results as patients carrying *GSTP*1 rs1695 polymorphism had shorter dexamethasone treatment duration, although they required longer hospitalization and oxygen supplementation. Some previously published studies also found associations between different *GST*s polymorphisms and higher COVID-19 infection rate, morbidity and/or mortality (Abbas et al., 2021; Coric et al., 2021). We did not detect or verify these associations, as our cohort included only patients with severe and critical disease, but not with mild or moderate disease or controls without COVID-19. Therefore, our results are not directly comparable with these reports.

Our findings regarding *NR3C1* polymorphisms are in accordance with the published literature. All three investigated *NR3C1* polymorphisms were previously reported to be associated with glucocorticoid resistance (Sæves et al., 2012; Gasic et al., 2018; Pahor et al., 2021), and were associated with longer hospitalization and/or oxygen requirement in our study. *NR3C1* rs6198 (c.*3833A>G) is located within the ATTTA motif (forward DNA strand) in exon 9β, which is part of mRNA stabilization region. The change from ATTTA to GTTTA results in altered mRNA stability of GRβ isoform, resulting in increased GRβ expression and stability. GRβ has an inhibitory effect on the active form GRα, leading to glucocorticoid resistance (Sæves et al., 2012; Gasic et al., 2018; Pahor et al., 2021). Several findings of the present study are in accord with this mechanism. Namely, patients carrying *NR3C1* rs6198 polymorphism had a shorter treatment with dexamethasone, and at the same time, the therapy was often switched from dexamethasone to methylprednisolone. Furthermore, these patients required longer oxygen supplementation and were hospitalized for a longer time.

We also investigated two *NR3C1* polymorphisms, rs33389 and rs33388, located in intron 2, in the region where alternative splicing takes place. The presence of rs33389T allele and rs33388 A allele leads to increased GRγ isoform expression (Gross et al., 2009). The latter has similar affinity for GCs as GRα, but with lower stability for binding to GCs responsive elements (GREs) in DNA, resulting in poor GCs response (Beger et al., 2003)*.* However, some other studies suggested that the *NR3C1* rs33389 C allele and *NR3C1* rs33388 T allele form parts of the rs41423247–rs33389–rs33388 ACT haplotype which is associated with glucocorticoid enhanced sensitivity (Stevens et al., 2004; Gasic et al., 2018). In our study, patients carrying rs33388 AA genotype had higher requirement for ICU treatment, needed longer hospitalization and tended to have higher odds for switching treatment from GCs to methylprednisolone. When we performed the haplotype analysis for the three *NR3C1* polymorphisms investigated in our study, rs6198–rs33388–rs33389 CAT haplotype was associated with duration of hospitalization, duration of oxygen supplementation, and switching from dexamethasone to methylprednisolone, but not with the duration of dexamethasone treatment.

We have also observed an interesting pattern regarding the associations of *NR3C1* polymorphisms with duration of hospitalization and oxygen supplementation as they were not significant in univariate model, but showed nominally or statistical significance after adjustment for chronic diseases and/or age. Several studies reported that *NR3C1* polymorphisms, rs6198 G allele in particular, were associated with dysregulated hypothalamic-pituitary-adrenal (HPA) axis (Szczepankiewicz et al., 2011; Fortier et al., 2013; Rovaris et al., 2013; Plieger et al., 2018). Prolonged exposure to physiological stress, e.g., chronic diseases, may also result in overstimulated HPA axis, and lead to higher plasma cortisol levels, which interfere with immunological and anti-inflammatory processes (Jones and Gwenin, 2021). Dysregulation of HPA axis has been proposed also in patients with COVID-19 with chronic diseases (Vassiliadi et al., 2021). The combination of these effects may explain why in our study patients with COVID-19 with chronic diseases and *NR3C1* polymorphism may have needed prolonged hospital care.

*ABCB1* gene encodes protein P-gp, which is a membrane transporter responsible for active transport of xenobiotics and endogenous substances from the cells. Genetic polymorphisms in the *ABCB1* gene may lead to altered activity and structure of P-gp, which can lead to unfavorable treatment outcome (Rychlik-Sych et al., 2018). *ABCB1* rs1045642 C and rs2032582 G alleles were reported to be associated with increased gene expression and function of P-gp that may have led to less effective treatment outcomes (Athanasoulia et al., 2012; Tsuji et al., 2013). In our study, *ABCB1* rs1045642 showed no significant associations, but the presence of one *ABCB1* rs2032582 G allele had a protective role in switching treatment to methylprednisolone. Regarding *ABCB1* rs1128503, the T allele has been so far associated with lower P-gp activity, which can result in ADRs (Vivona et al., 2014; Biswas, 2021). In our study, both homozygotes had longer hospitalization compared to heterozygotes. Longer hospitalization in TT homozygotes could be in part due to the occurrence of ADRs, leading to additional and longer medical care. However, the *ABCB1* rs1128503 T allele was in our results also associated with longer dexamethasone treatment, which suggests a contradiction with the before mentioned ADRs occurrence. Conflicting results regarding the role of this polymorphism were also reported in other infectious diseases. In children infected with HIV-1, an association was found between *ABCB1* rs1128503 CT heterozygotes and lower lopinavir plasma level when compared to homozygotes TT (Bellusci et al., 2013). However, a study on healthy adult volunteers detected no association between altered lopinavir plasma level and this polymorphism (la Porte et al., 2007). To the best of our knowledge, ours is the first study that links *ABCB1* polymorphisms with the treatment outcome of patients with COVID-19, although some ambiguities about the effect of these polymorphisms on the transport capacity of the ABC transporter still remain. Our results further support the observations of Bellusci et al. (2013) and Rychlik-Sych et al. (2018) suggesting that currently there are no uniform explanations about the impact of *ABCB1* polymorphisms. Furthermore, Rychlik-Sych et al. also indicated that due to the linkage disequilibrium between these polymorphisms, *ABCB1* haplotypes rather than individual polymorphisms might have a more important role, therefore our results should be further supported by additional studies.

The haplotype analysis strongly supported our observation about importance of *NR3C1* rs6198, *ABCB1* rs2032582, *GSTP1* rs1695 and especially *CYP3A4* rs35599367, additionally suggesting that these polymorphisms are good candidate for predictive biomarkers. The presence of polymorphic allele of *NR3C1* rs6198, *ABCB1* rs2032582 or *GSTP1* rs1695 polymorphism could predict poorer course of COVID-19 and also poorer treatment outcome. However, the best candidate for biological marker turned out to be *CYP3A4* rs35599367. It was associated with worse disease course and unfavorable outcome of COVID-19, both in the analysis of SNPs and in the analysis of haplotypes.

When interpreting our results, we have to keep in mind some limitations of our study. Our cohort included 107 patients, because we have included only patients hospitalized between September 2020 and December 2021. Thus, our study included only individuals who were hospitalized when, depending on the time of inclusion in the study, SARS-CoV2-2 B.1.258.17, Alpha or Delta variants were prevalent in the population, and we have stopped the recruitment before the more infectious omicron variant, which causes a milder course of the disease with a lower risk of death, became the predominant variant in the Slovenian population (Janezic et al., 2023). Although our patient cohort could be considered of a moderate size, a bigger cohort could give some clearer answers. As many of our results are only nominally and not statistically significant, we interpreted the results with caution to avoid over-interpretation. Also, our study would benefit from some additional clinical information, such as ADRs occurrence and reasons for treatment switching. These data were available only for some patients, and although they indicated that shorter duration of dexamethasone treatment was mostly linked to switching the treatment to methylprednisolone, we did not include them in the statistical analysis as it would be too biased due to the large number of missing data. Nevertheless, despite all its limitations, this study provides many new insights into the genetic factors that may influence the course of the disease and the treatment outcomes in patients with COVID-19.

## 5 Conclusion

In conclusion, our main findings suggest the impact of *CYP3A4* rs35599367 polymorphism on the occurrence of critical COVID-19 and also on poor outcome of dexamethasone treatment. *NR3C1* rs6198 polymorphism was associated with worse treatment outcomes, while *NR3C1* rs33388 was associated with better treatment outcomes. These identified polymorphisms hold potential for personalized medicine approaches. With the preemptive stratification of patients, we could predict the course of the disease and the response to treatment, and thus adapt treatment and care to the individual’s needs.

## Data Availability

The original contributions presented in the study are included in the article, further inquiries can be directed to the corresponding author.
