# Supplementary material for: Genetic variability in the glucocorticoid pathway and treatment outcomes in hospitalized patients with COVID-19: a pilot study
Source: Front Pharmacol. 2024 Jul 29;15:1418567. doi: 10.3389/fphar.2024.1418567 (PMC11317398; doi:10.3389/fphar.2024.1418567)
Supplement: Supplementary file 1 [file Table1.pdf]

Supplementary information:

Genetic variability in the glucocorticoid pathway and treatment outcomes in hospitalized patients with COVID-19: a pilot study

P. Štampar[1], T. Blagus[1], K. Goričar[1], P. Bogovič[2], G. Turel[2], F. Strle[2], V. Dolžan[1]

[1]Pharmacogenetics Laboratory, Institute of Biochemistry and Molecular Genetics, Faculty of Medicine, University of Ljubljana, Ljubljana, Slovenia

[2]Department of Infectious Diseases, University Medical Centre Ljubljana, Ljubljana, Slovenia

Supplementary Table 1: Genotype frequency and characteristics of studied SNPs

Gene	Polymorphisms	Location in gene (Sherry et al., 2001;Harrison et al., 2024)	MAF (Sherry et al., 2001)	Predicted polymorphisms function	Genotype	N (%)	Frequency of polymorphic allele	HWE equilibrium p-value
NR3C1	rs6198 [1] c.*3833A>G	Coding Sequence Variant 3'UTR* exon 9b	0.17	Affects mRNA stability (Gasic et al., 2018)	TT	77 (72.6)	0.16	0.290
					TC	25 (23.6)		
					CC	4 (3.8)		
	rs33388 c.1185-3562T>A	Intron variant	0.45	Affects splicing (Gasic et al., 2018)	AA	29 (27.1)	0.48	0.905
					AT	54 (50.5)		
					TT	24 (22.4)		
	rs33389 c.1185-6766G>A	Intron variant	0.15	Affects splicing (Gasic et al., 2018)	CC	24 (22.4)	0.52	0.905
					CT	54 (50.5)		
					TT	29 (27.1)		
ABCB1	rs1045642 p.Ile1145=	Coding Sequence Variant Exon 26	0.48	Affects splicing	TT	27 (25.2)	0.48	0.371
					CT	58 (54.2)		
					CC	22 (20.6)		
	rs1128503 p.Gly412=	Coding Sequence Variant Exon 12	0.58	Affects splicing	TT	18 (16.8)	0.59	0.970
					CT	52 (48.6)		
					CC	37 (34.6)		
	rs2032582 [17] p.Ala893Ser/Thr	Coding Sequence Variant Exon 21		Missense Variant	GG	34 (37.8)		0.337
					GT	39 (43.3)		
			0.45 (T)		TT	12 (13.3)	0.38 (T)	
			0.001 (C)		TA	5 (5.6)	0.03 (A)	
GSTP1	rs1695 p.Ile105Val	Coding Sequence Variant	0.33	Affects splicing	TT	18 (16.8)	0.59	0.899

		exon 5			TC	51 (47.7)		
					CC	38 (35.5)		
	rs1138272 p.Ala114Val	Coding Sequence Variant exon 6	0.07	Affects splicing	CC	89 (83.2)	0.09	0.852
					CT	17 (15.9)		
					TT	1 (0.9)		
GSTM1	*deletion*	Coding Sequence Variant		Affects enzyme activity (Gasic et al., 2018)	no deletion	52 (48.6)		
					deletion	55 (51.4)		
GSTT1	*deletion*	Coding Sequence Variant		Affects enzyme activity (Gasic et al., 2018)	no deletion	83 (77.6)		
					deletion	24 (22.4)		
CYP3A4	rs35599367 c.522-191C>T	Intron Variant	0.05	Affects mRNA stability (Wang and Sadee, 2016)	CC	101 (94.4)	0.03	0.765
					TC	6 (5.6)		
					TT	0 (0)		
	rs2740574 g.442C>T	2KB Upstream Variant	0.03	Affects transcription factor binding site	AA	103 (96.3)	0.02	0.844
					GA	4 (3.7)		
					GG	0 (0)		
CYP3A5	rs776746 [2] c.6986G>A	Non coding transcript variant	0.05	Affects splicing	CC	91 (85)	0.08	0.581
					TC	14 (13.1)		
					TT	1 (0.9)		
	rs10264272 [10] p.Lys208=	Coding Sequence Variant	1	Affects splicing	CC	0 (0)	1	/
					CT	0 (0)		
					TT	95 (100)		

[ ] number of missing data

References

Gasic, V., Zukic, B., Stankovic, B., Janic, D., Dokmanovic, L., Lazic, J., et al. (2018). Pharmacogenomic markers of glucocorticoid response in the initial phase of remission induction therapy in childhood acute lymphoblastic leukemia. *Radiol. Oncol.* 52 (3)**,** 296-306. doi: 10.2478/raon-2018-0034.

Harrison, P.W., Amode, M.R., Austine-Orimoloye, O., Azov, A.G., Barba, M., Barnes, I., et al. (2024). Ensembl 2024. *Nucleic Acids Res.* 52(D1)**,** D891-d899. doi: 10.1093/nar/gkad1049.

Sherry, S.T., Ward, M.H., Kholodov, M., Baker, J., Phan, L., Smigielski, E.M., et al. (2001). dbSNP: the NCBI database of genetic variation. *Nucleic Acids Res.* 29(1)**,** 308-311. doi: 10.1093/nar/29.1.308.

Wang, D., and Sadee, W. (2016). CYP3A4 intronic SNP rs35599367 (CYP3A4*22) alters RNA splicing. *Pharmacogenet Genomics.* 26 (1)**,** 40-43. doi: 10.1097/fpc.0000000000000183
